# Effects of Cellular Methylation on Transgene Expression and Site-Specific Integration of Adeno-Associated Virus

**DOI:** 10.3390/genes8090232

**Published:** 2017-09-18

**Authors:** Diptiman Chanda, Jonathan A. Hensel, Jerome T. Higgs, Rajat Grover, Niroop Kaza, Selvarangan Ponnazhagan

**Affiliations:** Department of Pathology, University of Alabama at Birmingham, Birmingham, AL 35294, USA; diptimanchanda@uabmc.edu (D.C.); jonathanhensel@uabmc.edu (J.A.H.); jthiggs@uab.edu (J.T.H.); grover@uab.edu (R.G.); kaza@uab.edu (N.K.)

**Keywords:** adeno-associated virus, 5-Azacytidine, gene expression, site-specific integration

## Abstract

DNA methylation is a major epigenetic event that affects not only cellular gene expression but that also has the potential to influence bacterial and viral DNA in their host-dependent functions. Adeno-associated virus (AAV) genome contains a high degree of CpG sequences capable of methylation in its terminal repeat sequences, which are the sole elements retained in AAV-based vectors used in gene therapy. The present study determined the influence of methylation status of the host cell on wild type (wt) AAV integration and recombinant (r) AAV transgene expression in HeLa cells. Results of the study indicated that hypo-methylation significantly enhanced both wtAAV chromosomal integration and transgene expression of rAAV. A direct influence of methylation on AAV integration was further confirmed by methylating the AAVS1 integration sites prior to viral infection with DNA trans-complementation assay. These results signify the importance of epigenetic status of target cells as one of the key factors in long-term transgene expression in AAV gene therapy.

## 1. Introduction

Success of any gene therapy application depends on long-term and robust expression of the transgene, which is often limited due to host defense mechanisms against viral infection. Evidence suggests suppression of viral gene expression in eukaryotic cells via DNA methylation and histone modification [1]. Un-methylated CpG regions of microbial DNA are immunogenic and are also targets for DNA methyltransferase (DNMT) activity and recruitment of polycomb and other protein complexes, resulting in repression of viral replication and transgene expression [2,3,4,5]. Similar epigenetic silencing has been reported in both retroviral and adenoviral vector infections [6,7]. Restoration of the silenced retroviral genome in different tissues of mice has been reported following treatment of a hypo-methylating agent, 5-Aza-2′deoxycytidine (5-Aza) [8]. Adeno-associated virus (AAV) is a non-pathogenic parvovirus currently being tested in many clinical gene therapy applications [9]. Wild type (wt) AAV-2, has anti-oncogenic properties against in Human Papillomavirus (HPV)-induced carcinogenesis [10]. There are at least four CpG islands in the 4.6 kb AAV genome that are larger than 400 base-pairs each [11]. They are located between the nucleotides, 199–2038 (1840 bp), 2076–2740 (665 bp), 2946–3786 (841 bp) and 3994–4430 (437 bp). The p5, p19 and the p40 promoters, which are responsible for the expression of replication (Rep) and capsid (Cap) proteins, are located within the first CpG island, suggesting the possibility that alteration in host-cell methylation status could impact wtAAV integration. Further, as in other gene therapy vectors, most of the recombinant (r) AAV vectors commonly employ cytomegalovirus immediate early gene promoter (CMV) for transgene expression, which also contains 3 CpG islands of more than 100 bp length. Therefore, methylation status of the target cell could affect AAV transgene expression.

Site-specific integration into human chromosome 19 is a major hallmark of wtAAV and studies have attempted induction of site-specific integration of rAAV for safe and long-term expression of therapeutic transgenes [12]. Since AAV Rep proteins are responsible for stable integration, integration of rAAV can be achieved by providing Rep function in trans. Previous studies have demonstrated that the leading 510 nucleotides at the 5′ end of AAVS1 sequence in the human chromosome 19 are a key to AAV integration, and contain sequence homology to the rep binding site (RBS) and the terminal resolution site (TRS) of AAV [13]. When the first 1020 base pairs of the AAVS1 were sequenced, analysis indicated that the 510 nucleotides are a part of a 662 base pair CpG island, starting at 203 nucleotides and ending at 864 nucleotides [11]. CpG islands are widely distributed in the mammalian genome and are typically methylated at cytosine bases. Un-methylated CpGs are located primarily in the 5′ regulatory region of active genes, and the addition of methyl groups may lead to silencing of gene expression [14].

There is a wide range of variability of transduction efficiency among different AAV serotypes and target cells. Slower rate of second strand synthesis, neutralizing antibodies and proteasome activation are reported to have negative effects on AAV transgene expression [15,16,17], but no study has specifically addressed the methylation status of target cells in AAV transduction. The present study evaluated the role of DNA methylation in AAV transgene expression and integration and determined that hypo-methylation of target cells affects site-specific integration of wtAAV and also transgene expression of rAAV. These results may have a significant impact on the application of AAV-based vectors in gene therapy.

## 2. Materials and Methods

### 2.1. Cell Lines and Reagents

HeLa cells were obtained from the American type culture collection (ATCC) (Manassas, VA, USA) and maintained in Dulbecco’s modified eagle’s medium (DMEM) supplemented with 10% new-born calf serum and penicillin-streptomycin (Mediatech Inc., Hendron, VA, USA). C18 cells, a derivative of human embryonic kidney cell line 293, expressing Epstein-Barr virus nuclear antigen (EBNA-1), were purchased from ATCC and maintained in DMEM supplemented with 10% fetal bovine serum and antibiotics. Detroit-6 cells (latently infected with AAV-2 and harboring integrated copies of the viral genome in AAVS1) were a generous gift from Dr. Robert Kotin, NIH, Bethesda, MD, USA. 5-Aza cytidine (5-Aza; cat# A3656) was purchased from Sigma-Aldrich Corp. (St. Louis, MO, USA). N-hydroxysuccinimide ester (NHS)-Biotin (long-arm, cat# SP-1210) and Alexa Fluor594-labeled streptavidin were purchased from Vector Laboratories (Burlingame, CA, USA) and Molecular Probes Inc. (Eugene, OR, USA) respectively. The P220.2 plasmid, containing 8.2 kb human AAVS1 sequence, was kindly provided by Dr. Kenneth Berns, University of Florida, USA. Advantage high fidelity 2 PCR kit was purchased from (cat# 639123, Clontech Laboratories Inc., Mountain View, CA, USA) and Vybrant^®^ 3-(4,5-dimethylthiazol-2-yl)-2,5-diphenyltetrazolium bromide (MTT) Cell Proliferation Assay Kit was purchased from Molecular Probes, Inc. and used according to manufacturer’s instructions.

### 2.2. AAV Production and Purification

Packaging and purification of both wild type AAV and recombinant AAV-GFP in serotype 2 capsids were performed in adenovirus-free system as described previously [18]. Particle titers of each of the purified viruses were determined by quantitative slot-blot analysis [18].

### 2.3. Polymerase Chain Reaction(PCR) Analysis

AAV-AAVS1 junction fragments were amplified using two rounds of PCR reactions following a published protocol [19]. Briefly, first amplifications were carried out with one AAV-inverted terminal repeat (AAV-ITR) targeted, forward primer (5′-GTAGCATGGCGGGTTAATCA-3′) and one AAVS1 targeted, reverse primer (5′-GCGCGCAGAAGCCAGTAGAGC-3′) in a total volume of 50 μL. The PCR reactions were 5 min denaturation at 94 °C, followed by 25 cycles of 30 s each at 94 °C denaturation, 2 min annealing at 68 °C, and 2 min extension at 68 °C. Ten μL of the amplification product was then re-amplified for 25 cycles using similar thermocycler conditions, but excluding the first incubation for 5 min at 94 °C. Primers used for second round of amplification were: ITR-specific forward primer, 5′-TTAACTACAAGGAACCCCTAGTGATGG-3′ and AAVS1-specific reverse primer 5′-GATAGACCAGACCTGAGCTATGGGAG-3′, designed to target AAV-ITR and AAVS1 sequences within the first amplification product.

### 2.4. Southern Hybridization

The DNA samples were separated on 1–1.5% agarose gels and transferred to Gene Screen Plus membrane (PerkinElmer Life and Analytical Sciences, Boston, MA, USA). Hybridizations were performed using P^32^-labeled probes corresponding to CMV promoter, AAV and AAV-AAVS1 junction fragment respectively following protocols described earlier [20].

### 2.5. Biotinylation and Streptavidin Labeling of rAAV-2

Biotin labeling of AAV was performed following protocol described earlier [21]. Briefly, 1 × 10^10^ rAAV-2-GFP particles were incubated with 5 μg of water-soluble NHS-biotin per mL in a volume of 50 μL HEPES buffer for 1 h at room temperature. Free biotin was removed using a Centricon-30 molecular weight cut-off filter. HeLa cells were infected with NHS-biotin-labeled rAAV-2 at a ratio of 1000 vector genome (vg)/cell, following 72 h of 5-Aza treatment in 8-well chambered slides. Cells were incubated with the virus for 1 h at 4 °C for binding of the viral particles to the cell membrane. After 1 h, cells were washed in serum-free medium two times and either fixed in 2% paraformaldehyde or continued at 37 °C for further infection in the presence of medium containing 10% serum. Following fixation, cells were washed with sterile phosphate buffered saline + Tween 20 (PBST) and incubated with 10 μg/mL Alexa-fluor-594 labeled streptavidin in PBST for 30 min. For analyzing the intracellular trafficking of rAAV, HeLa cells were treated with 0.01% Triton-X-100 (Sigma-Aldrich) post-fixation for permeabilization of streptavidin. Excess streptavidin was washed off with PBST and slides were mounted in Vectashield^®^ mounting medium from Vector Laboratories, visualized and imaged in a Zeiss LSM 710 Laser Confocal Scanning Microscope (Carl Zeiss Microscopy GmbH, Jena, Germany) using 0.42 μm resolution and Zen2008 4.7.2 software (Carl Zeiss). All in vitro experiments were repeated at least twice.

## 3. Results

### 3.1. Effect of Hypo-Methylation on AAV-Transgene Expression

**Figure 1:**
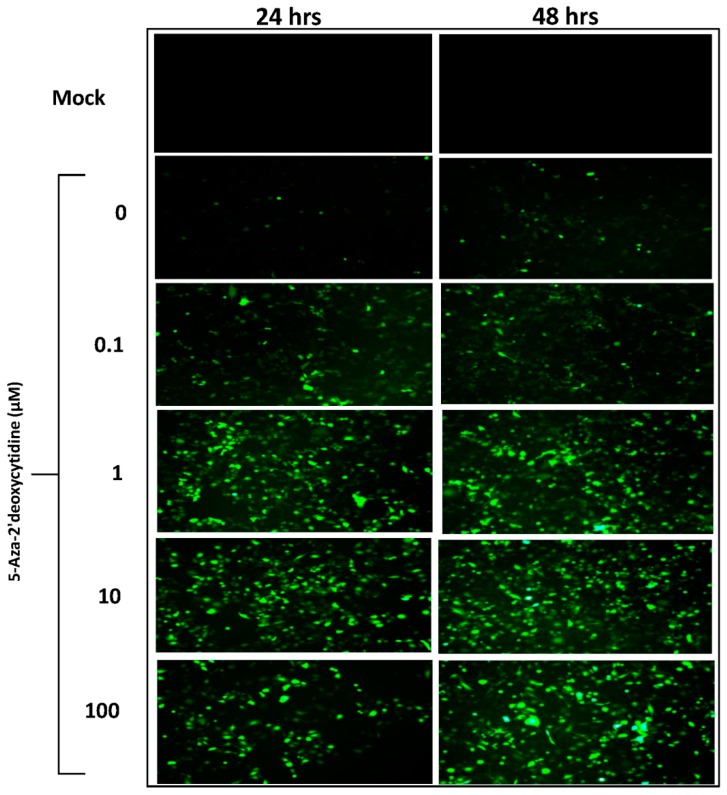
Effects 5-Aza on Adeno-associated virus (AAV)-transgene expression (green fluorescent protein; GFP) in HeLa cells. HeLa cells were maintained in various concentration of 5-Aza for 72 h, when they were infected with recombinant (r) AAV-GFP. GFP expression was compared between untreated and 5-Aza treated HeLa cells after 24 and 48 h under a fluorescence microscope (Original magnification 200×).

5-Aza-2′ deoxycytidine (5-Aza) incorporates into the cellular DNA and inhibits DNA methyltransferase (DNMT1) activity following covalent binding of the cytosine-C5 methyltransferase to 5-Aza residues in the CpG sites of the DNA [22]. This results in progressive demethylation of the DNA and gene activation. First, cytotoxic effect of 5-Aza on HeLa cells was tested by maintaining HeLa cells at various concentrations (0.1 μM, 1 μM, 10 μM and 100 μM) of 5-Aza for 72 h followed by cell proliferation analysis by MTT assay. Results of this study indicated a significant decrease in HeLa cell viability at 5-Aza concentrations at and above 10 μM (data not shown). To determine the influence of hypo-methylation on AAV transgene expression, low confluent Hela cells were cultured in various concentrations of 5-Aza for 72 h. Cells were then transduced with rAAV-2-GFP (10 vector genome containing particles/cell) and continued in the culture with 5-Aza. After 24 h, GFP expression was compared between the treatment groups. Significantly high expression of GFP was observed in HeLa cells treated with 5-Aza, suggesting hypo-methylation enhances AAV transgene expression (Figure 1). 1 μM 5-Aza showed the highest GFP expression whereas no further increase in GFP expression was observed with 10 and 100 μM 5-Aza. Therefore, we adhered to 0.1 μM and 1 μM 5-Aza concentrations in the following experiments.

### 3.2. Enhanced GFP Expression in rAAV-GFP Infected Hypo-Methylated HeLa Cells Is Not Due to Enhanced Viral Transduction Following Aza Treatment

**Figure 2:**
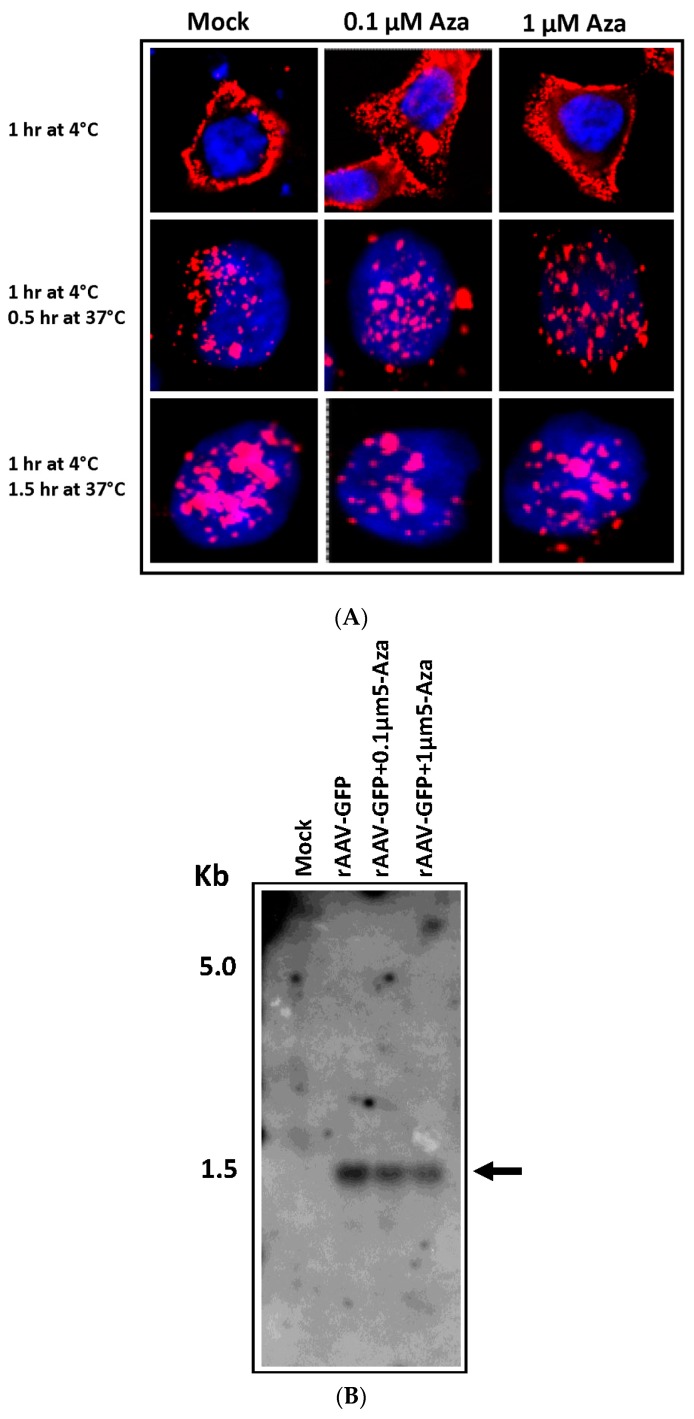
Intracellular trafficking of rAAV-2 following Aza treatment (**A**). HeLa cells were treated with 0.1 μM and 1 μM Aza for 72 h and followed by infection with AAV, labeled with NHS (N-hydroxysuccinimide ester)-Biotin. Cells were fixed in paraformaldehyde following virus binding at 4 °C for 1.5 h and 90 min at 37 °C after virus binding. Membrane bound and internalized biotinylated AAV particles were detected using Alexa-fluor 594 labeled streptavidin and visualized and imaged in a laser scanning confocal microscope (Original magnification 400×); (**B**) Southern blot detection of low molecular weight DNA obtained from 5-Aza treated and untreated HeLa cells showing comparable amount of monomeric rAAV-GFP. The membrane was probed with a P^32^-labeled Cytomegalovirus (CMV) probe. The original ethidium bromide stained gel is provided to show loading of samples.

To determine if pre-treatment of HeLa cells with 5-Aza resulted in an increase of vector entry or intracellular trafficking, HeLa cells were grown on 8-chambered culture slides and infected with NHS-biotin-labeled rAAV-GFP following 72 h of Aza treatment. Detection of membrane-bound and internalized viral particles was performed using Alexa Fluor 594-labeled streptavidin. Results indicated no significant difference in viral uptake between Aza-treated and untreated HeLa cells, suggesting 5-Aza treatment does not influence viral entry (Figure 2A). Further, to quantitatively determine if such variation exists in vector entry or intra-cellular trafficking of AAV, low molecular weight DNA was isolated by Hirt method [23], 48 h following rAAV-GFP transduction of untreated or 1 μM 5-Aza-treated HeLa cells. The relative vector genome was analyzed by Southern Blot using a P^32^-labeled DNA probe corresponding to the CMV promoter. Results of this analysis also indicated no significant difference between untreated and 5-Aza treated cells, further confirming that effects of hypo-methylation do not affect AAV internalization (Figure 2B).

### 3.3. Effect of Hypo-Methylation on Site-Specific Integration of wtAAV

**Figure 3:**
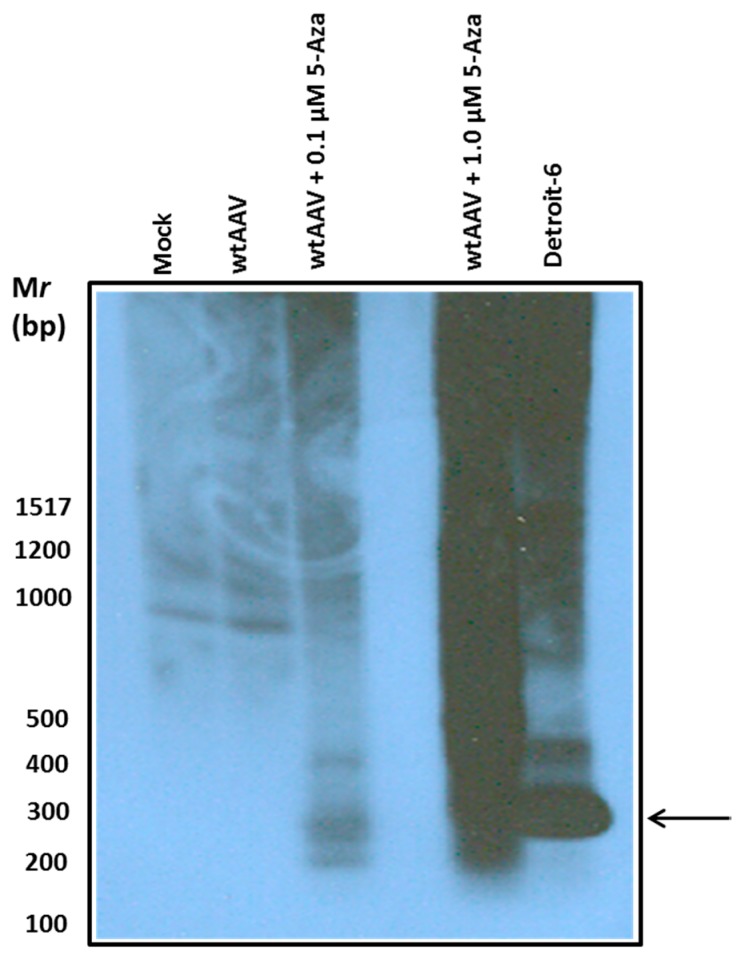
Effect of 5-Aza on wtAAV site-specific integration. Genomic DNA was isolated from control HeLa cells and wt AAV infected HeLa cells following indicated concentrations of 5-Aza treatment. 1 μg of genomic DNA from above were subjected PCR analysis for integration. PCR products were separated on a 1.5% DNA gel and transferred to a nylon membrane. The membrane was probed with a P^32^-labeled junction fragment probe. DNA from Detroit-6 cells was used as a control for the polymerase chain reaction (PCR) reaction. The smearing pattern of the PCR products is due multiple AAV integration events within the AAVS1 region, following treatment with 1 μM 5-Aza. The arrow indicates the expected size of AAV-AAVS1 integration junction fragment after PCR amplification.

In order to determine if hypo-methylation would influence wtAAV2 site-specific integration into AAVS1, HeLa cells at very low confluency were cultured at various concentrations of 5-Aza (0.1 μM and 1 μM) for 72 h and infected with wtAAV (1000 vg/cell). The cells were maintained in culture for 96 h in the presence of 5-Aza before harvesting. Genomic DNA was isolated and 1 μg of genomic DNA was subjected to nested-PCR reactions using primers to determine integration as described in the materials and methods. The PCR products were resolved on a 1.5% agarose gel, transferred to a nylon membrane and hybridized with a P^32^-labeled junction fragment probe, which is derived from the AAV integration junction sequence of Detroit-6 cells. Results of this analysis (Figure 3) indicated a significant increase in integration events following 5-Aza treatment, suggesting hypo-methylation may lead to incorporation of more copies of AAV genome into AAVS1. 1 μM 5-Aza led to the highest amount of integration as compared to untreated HeLa cells.

### 3.4. Hypo-Methylation Increases Site-Specific Integration of wtAAV Affecting AAVS1

**Figure 4:**
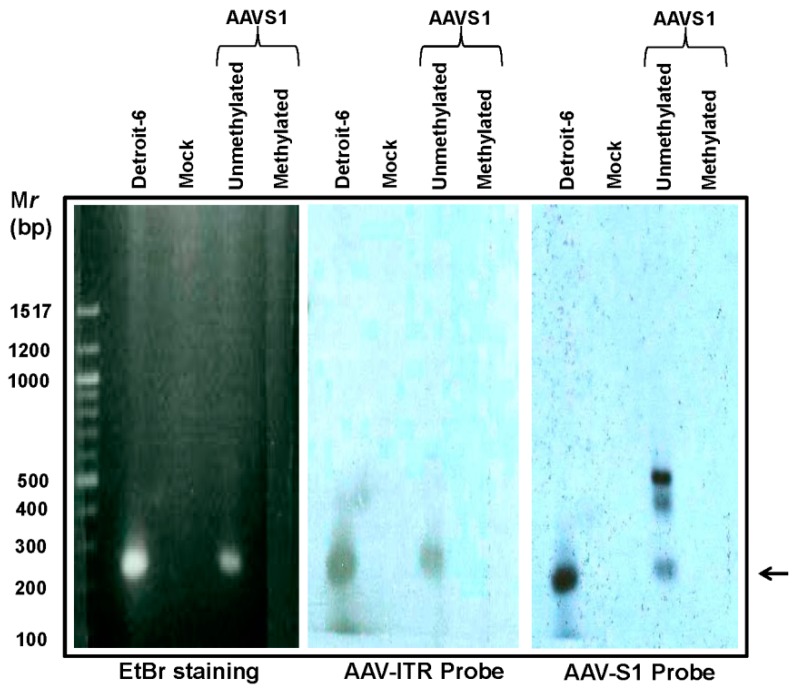
Effect of de novo AAVS1 methylation on wtAAV site-specific integration. C18 cells were transfected with unmethylated and in vitro methylated p220.2 plasmids containing the 8.2 kb human AAVS1 and infected with wtAAV. Low molecular DNA was isolated by Hirt method [23] from mock, unmethylated and methylated AAVS1 transfected C18 cells and subjected to nested-PCR analysis. PCR products were separated by electrophoresis and subjected to Southern hybridization using a P^32^-labeled probe against AAV-inverted terminal repeat (ITR) and AAVS1. DNA from Detroit-6 cells was used as a control.

Since AAV and AAVS1 share similarities in sequence and CpG enrichment, we sought to determine if the increased integration frequency observed above could be enhanced due to the effects from hypomethylation of AAVS1. To this end, we performed a trans-complementation assay in HeLa cells where an EBV-based shuttle vector (p220.2) containing the 8.2 kb AAVS1 sequence was first methylated using CpG methylase and a methyl donor, S-adenosyl methionine (SAM), and then transfected into C18 cells using lipofectamine. Control C18 cells were transfected with unmodified p220.2-AAVS1 plasmid. Then, 24 h after transfection, the cells were infected with wild-type AAV (1000 viral particles/cell) and low molecular weight DNA was extracted 96 h after the infection. To determine the effect on integration, a nested PCR was performed using two sets of primers flanking the AAV right -ITR region and AAVS1 sequence to compare AAV integration between unmethylated and methylated AAVS1. Genomic DNA isolated from Detroit-6 cells was used as a positive control. The PCR products were run on 1.5% agarose gel and transferred to a nylon membrane and confirmed by Southern Hybridization using P^32^-labeled probes, complementary to AAV and AAVS1. Results of this study, shown in Figure 4, indicated a lack of AAV integration when AAVS1 sites were methylated.

## 4. Discussion

Targeted inactivation of foreign DNA is an evolutionary defense mechanism against bacteria and viruses. The numbers of CpG di-nucleotides are significantly less in eukaryotic viruses with genome size less than 30 kb [24]. HPV, adenovirus, hepatitis-B virus and AAV are exceptions to this rule and possess abundant CpGs [24]. These CpG islands are targets of host DNMTs, which inhibit viral replication and gene expression via DNA methylation and histone modification. 5-Aza incorporates into the cellular DNA during replication and inhibits DNA methyltransferase activity [22].

AAV is a single-stranded DNA virus whose replication is dependent on helper viruses such as adenovirus, herpes simplex virus or vaccinia virus [25]. Moreover, second-strand synthesis is rate-limiting for both AAV replication and transgene expression [26]. Our results indicated significantly higher transgene (GFP) expression in HeLa cells following 5-Aza treatment, which was independent of the number of AAV particles that actually entered the cells. It is likely that 5-Aza incorporated into the CpGs of the CMV promoter region of rAAV during the second strand synthesis which prevented epigenetic inactivation of rAAV-GFP, resulting in enhanced GFP expression.

AAV is reported to integrate in the chromosome with a very low frequency, and instead remains as episomal concatamers in the host nucleus [27]. Therefore, there is a high likelihood of elimination from the target cells during cell division. In this study, we sought to determine if 5-Aza could influence site-specific integration of wtAAV, capitalizing on the CpG rich nature of the AAVS1 site. The integration property of wtAAV2 in human chromosome 19, mediated by non-homologous recombination, has been well characterized [12]. Studies have characterized this domain in human chromosome 19, which contains homologous sequences to AAV Rep binding element and the TRS. Our results indicate a significantly higher integration of AAV into AAVS1 after 5-Aza treatment. It is apparent that 5-Aza treatment has resulted in the loss of methyl groups from the CpG rich AAVS1 site leading to integration of an increased number of AAV genomes. A smear pattern of the integration fragments was observed in the HeLa cells treated with 1 μM 5-Aza. Previous reports indicated that site-specific integration of AAV in AAVS1 can occur in a region spanning 500 bp, immediately following the RBS and TRS sites [19]. Therefore, a smear pattern of PCR amplification products suggest multiple sites of integration. In this study, we have also demonstrated significant inhibition of wtAAV integration following in vitro methylation and subsequent transfection of AAVS1 genomic sequence into the C18 cells. These findings suggest that cellular epigenetic status can be altered to enhance site-specific AAV integration, and therapeutic transgene expression.

Currently, AAV vectors are increasingly being tested for gene therapy of monogenic diseases, infectious diseases and cancer with modest success. Tumor cells are often hyper-methylated at the promoters of the tumor suppressor genes and over-express DNMTs. Inhibition of DNMT1, DNMT3a and DNMT3b has been reported to prevent tumor growth in many types of cancers [28]. Increased expression of these enzymes may also result in silencing of rAAV transgene expression or compromise AAV site-specific integration wherever applicable in target cells. Although 5-Aza is shown in vitro and in vivo to inhibit DNMT1 and enhance therapeutic response in many cancers, caution should be exercised as genome-wide inhibition of DNA methylation might activate certain oncogenes. Therefore, targeted inhibition of DNMTs or development of CpG-free promoters might be useful in enhancing the effects of AAV-based gene therapy applications.

## References

[1] Doerfler W. (2011). Epigenetic consequences of foreign DNA insertions: De novo methylation and global alterations of methylation patterns in recipient genomes. Rev. Med. Virol..

[2] Ryan J.H., Zolotukhin S., Muzyczka N. (1996). Sequence requirements for binding of rep68 to the adeno-associated virus terminal repeats. J. Virol..

[3] Krieg A.M. (1999). Direct immunologic activities of CpG DNA and implications for gene therapy. J. Gene Med..

[4] Bauer S., Kirschning C.J., Hacker H., Redecke V., Hausmann S., Akira S., Wagner H., Lipford G.B. (2001). Human Tlr9 confers responsiveness to bacterial DNA via species-specific CpG motif recognition. Proc. Natl. Acad. Sci. USA.

[5] Lynch M.D., Smith A.J., De Gobbi M., Flenley M., Hughes J.R., Vernimmen D., Ayyub H., Sharpe J.A., Sloane-Stanley J.A., Sutherland L. (2012). An interspecies analysis reveals a key role for unmethylated CpG dinucleotides in vertebrate polycomb complex recruitment. EMBO J..

[6] Pannell D., Ellis J. (2001). Silencing of gene expression: Implications for design of retrovirus vectors. Rev. Med. Virol..

[7] Brooks A.R., Harkins R.N., Wang P., Qian H.S., Liu P., Rubanyi G.M. (2004). Transcriptional silencing is associated with extensive methylation of the CMV promoter following adenoviral gene delivery to muscle. J. Gene Med..

[8] Jaenisch R., Schnieke A., Harbers K. (1985). Treatment of mice with 5-azacytidine efficiently activates silent retroviral genomes in different tissues. Proc. Natl. Acad. Sci. USA.

[9] Ortolano S., Spuch C., Navarro C. (2012). Present and future of adeno associated virus based gene therapy approaches. Recent Pat. Endocr. Metab. Immune Drug Discov..

[10] Hermonat P.L. (1994). Adeno-associated virus inhibits human papillomavirus type 16: A viral interaction implicated in cervical cancer. Cancer Res..

[11] Li L.C., Dahiya R. (2002). Methprimer: Designing primers for methylation pcrs. Bioinformatics.

[12] Linden R.M., Ward P., Giraud C., Winocour E., Berns K.I. (1996). Site-specific integration by adeno-associated virus. Proc. Natl. Acad. Sci. USA.

[13] Giraud C., Winocour E., Berns K.I. (1994). Site-specific integration by adeno-associated virus is directed by a cellular DNA sequence. Proc. Natl. Acad. Sci. USA.

[14] Deaton A.M., Bird A. (2011). CpG islands and the regulation of transcription. Genes Dev..

[15] McCarty D.M. (2008). Self-complementary AAV vectors; advances and applications. Mol. Ther..

[16] Finn J.D., Hui D., Downey H.D., Dunn D., Pien G.C., Mingozzi F., Zhou S., High K.A. (2010). Proteasome inhibitors decrease AAV2 capsid derived peptide epitope presentation on MHC class I following transduction. Mol. Ther..

[17] Mingozzi F., High K.A. (2011). Immune responses to AAV in clinical trials. Curr. Gene Ther..

[18] Zolotukhin S., Byrne B.J., Mason E., Zolotukhin I., Potter M., Chesnut K., Summerford C., Samulski R.J., Muzyczka N. (1999). Recombinant adeno-associated virus purification using novel methods improves infectious titer and yield. Gene Ther..

[19] Lamartina S., Roscilli G., Rinaudo D., Delmastro P., Toniatti C. (1998). Lipofection of purified adeno-associated virus rep68 protein: Toward a chromosome-targeting nonviral particle. J. Virol..

[20] Ren C., Kumar S., Shaw D.R., Ponnazhagan S. (2005). Genomic stability of self-complementary adeno-associated virus 2 during early stages of transduction in mouse muscle in vivo. Hum. Gene Ther..

[21] Ponnazhagan S., Mahendra G., Kumar S., Thompson J.A., Castillas M. (2002). Conjugate-based targeting of recombinant adeno-associated virus type 2 vectors by using avidin-linked ligands. J. Virol..

[22] Christman J.K. (2002). 5-azacytidine and 5-aza-2′-deoxycytidine as inhibitors of DNA methylation: Mechanistic studies and their implications for cancer therapy. Oncogene.

[23] Hirt B. (1967). Selective extraction of polyoma DNA from infected mouse cell cultures. J. Mol. Biol..

[24] Karlin S., Doerfler W., Cardon L.R. (1994). Why is CpG suppressed in the genomes of virtually all small eukaryotic viruses but not in those of large eukaryotic viruses?. J. Virol..

[25] Schlehofer J.R., Ehrbar M., zur Hausen H. (1986). Vaccinia virus, herpes simplex virus, and carcinogens induce DNA amplification in a human cell line and support replication of a helpervirus dependent parvovirus. Virology.

[26] Ferrari F.K., Samulski T., Shenk T., Samulski R.J. (1996). Second-strand synthesis is a rate-limiting step for efficient transduction by recombinant adeno-associated virus vectors. J. Virol..

[27] McCarty D.M., Young S.M., Samulski R.J. (2004). Integration of adeno-associated virus (AAV) and recombinant AAV vectors. Ann. Rev. Genet..

[28] Luszczek W., Cheriyath V., Mekhail T.M., Borden E.C. (2010). Combinations of DNA methyltransferase and histone deacetylase inhibitors induce DNA damage in small cell lung cancer cells: Correlation of resistance with IFN-stimulated gene expression. Mol. Cancer Ther..

